# Editorial: Interactions of Plants With Bacteria and Fungi: Molecular and Epigenetic Plasticity of the Host

**DOI:** 10.3389/fpls.2020.00274

**Published:** 2020-03-04

**Authors:** Valentina Fiorilli, Marco Catoni, Luisa Lanfranco, Nicolae Radu Zabet

**Affiliations:** ^1^Department of Life Sciences and Systems Biology, University of Turin, Turin, Italy; ^2^School of Biosciences, University of Birmingham, Birmingham, United Kingdom; ^3^School of Life Sciences, University of Essex, Colchester, United Kingdom

**Keywords:** plant-bacterial interactions, plant-fungal interactions, molecular mechanisms, omics tools, genotypes

In both natural and agricultural environments plants live in association with a multitude of microorganisms belonging to different microbial types, mainly bacteria and fungi. Some of these microbes regulate positively plant growth and productivity, while others can damage the host with important ecological and economic consequences. During the last decades, many studies contributed to unravel the multifaceted process at the basis of plant microbe-interactions (Cheng et al., 2019). However, many issues remain still unsolved, as for example how plants could discriminate between beneficial and pathogenic microbes or between different pathogen attackers, or which gene regulatory networks are responsible for host-microbe interactions and their degree of conservation among species.

When challenged by pathogens, plants trigger highly complex defense system, in order to recognize invader organisms and translate this signal into defense such as the expression of defense response genes. This plant immune system relies on a wide variety of different strategies, showing high plasticity in the response depending on the attacker lifestyle. These processes involve the regulation of genes, small RNAs, signal molecules, plant hormones, which can act locally or systemically through plant organs.

On the other hand, plants are strictly associated with microbial symbionts in natural environments, often poor in nutrients (van der Heijden et al., 2008). Beneficial microbes in the soil could help the host to overcome the nutritional and abiotic stresses, boost plant growth and fitness, and sustain plant productivity. In addition, beneficial associations can enhance the defensive ability of plants, resulting in faster and stronger defense activation upon pathogens attacks. While these phenomena have been widely described, the underling molecular mechanisms remain elusive. Changes in transcription, protein regulation and phytohormones accumulation have been reported during plant-beneficial microbe interactions. Moreover, epigenetic modifications triggering stable changes in plant's transcriptional capacity are emerging as relevant modulator of plant's responses to microbes, with potential role in memory and priming (Alonso et al., 2019).

With 13 original contributions, this Research Topic provides an overview of the current state of the art on the field of plant microbe-interactions. This Topic includes a combination of Reviews, Mini Reviews and Original Research Articles, focused on the role of the molecular infrastructures evolved by plants to manage different microbe-interactions, revealing that a complex plant–microorganism genotype and environment combinations could determine the outcome of the interaction.

Recently, genome wide RNA sequencing has become a popular approach to study transcriptional changes also in non-model organisms. Yuan et al. investigated the increased growth and sesquiterpenoids accumulation induced by the endophyte *Gilmaniella* in the medicinal herb *Atractylodes lancea* combining transcriptomic (RNAseq) and proteomic approaches. Authors observed that the presence of the endophyte induced in the host suppression of genes involved in plant immunity and signaling, while genes involve in both primary and secondary metabolism such as phenylpropanoid and zeatin biosynthesis were upregulated.

An emerging theme is the significance of genetic variation in differentiating the biological response to harmful and beneficial microorganisms. In this Research Topic a number of contributions addressed the role of different host genotypes in plant-microbe interactions. Czembor et al. studied 98 maize inbreed lines, historically used in Poland, in relation to resistance to *Fusarium*, across a 2 year in-field experiment. They coupled HPLC analysis for the detection of fumolisin content to NGS approach (ddRADseq) to infer genetic distances across the lines; they then correlate genetic distance with resistance to *Fusarium*. Authors observed large differences in resistance and fumolisin accumulation across the lines and concluded that old lines represent a valuable source of resistant traits against *Fusarium*.

Another emerging and powerful approach to identify novel genes involved in plant-microbe interactions is represented by Genome-Wide Association (GWA) studies. The contribution of plant genetic variability to the positive effects of arbuscular mycorrhizal (AM) symbiosis have been investigated in different crops (Diedhiou et al., 2016; Lehnert et al., 2017; Watts-Williams et al., 2019), and, in this line, Davidson et al. tested the responsiveness to AM colonization of 334 rice cultivars inoculated with the AM fungus *Rhizophagus irregularis*. GWA mapping for hyphal colonization revealed 23 quantitative trait loci (QTLs) with putative impact on AM fungal colonization and identified candidate genes associated to three QTLs.

More specific topics on plant-pathogen interactions are addressed by Cao et al. who investigated the hypersensitive response (HR) against *Xanthomonas oryzae* pv. *oryzae* (*Xoo*) in rice mediated by genes ascribed to the major disease resistance pathway. By observing the physiological response to infection in the xylem parenchyma, authors concluded that dominant resistant genes mediate HR by prevalent autophagy-like cell death, while recessive genes induce HR by vacuole-mediated cell death.

Legumes and vegetables pathogens affect field-grown and greenhouse-grown crops worldwide. Two works investigated, in different host plants, the role of a key component of the plant disease-associated signal transduction pathway (Guo et al.) and a negative regulator of programmed cell death (PCD) process (Yan et al.). Both reports highlighted how manipulating the expression of a specific gene of interest, using a genetic approach, could determine the biological function of genes in the plant resistance process. These results pave the way for the identification of molecular targets potentially useful for breeding programs to control pathogen resistance in crops.

Two studies, Zhang H. et al. and Yang et al., focused on the study of the microbial partner, characterizing two genes which contribute to *Botrytis cinerea* virulence, one of the most notorious pathogenic species, in different host plants.

An emerging theme in the field of plant-microbe interactions is the relevance of mineral nutrients availability. Mineral nutrients are not only important for the growth and development of plants and microorganisms but they also play a key role in the dynamics and the outcome of plant -pathogenic/beneficial microbes interactions. Li et al. studied the association of the diazotrophic bacteria *Paenibacillus beijingensis* with wheat, maize and cucumber in conditions of high and low nitrogen in the soil, and they observed beneficial effects on plant growth related to improved nitrogen uptake and assimilation, in line to previous findings from other bacterial/host combinations (Xie et al., 2016; Hao and Chen, 2017). Zhang L. et al. reviewed current knowledge on the roles of plant aquaporins (AQPs) of the plasma membrane intrinsic protein family. The authors highlighted that AQPs are not only involved in maintaining the plant water or mineral nutrition status, but they also contribute to control plant immune system and pathogen susceptibility.

In nature, plants are very likely subjected to multiple complex interactions involving several organisms, rather than single bipartite relationships. Although less investigated, multiple interactions represent a system more similar to the natural environment, and their study can assist to select or design optimal bio-fertilization or bio-control procedures. In this Research Topic, different contributions addressed this issue. Abbasi et al. compared the ability of different plant growth promoting rhizobacteria (PGPR) and the application of a chemical fungicide to antagonize *Fusarium oxysporum* f. sp. *lycopersici* race 3 (*FOL*) in tomato plants. Interestingly, all bacterial treatments mitigated *FOL* disease symptoms at the same level or better than chemical treatments. In a mini-review, Miozzi et al. provided an overview of the impact of the arbuscular mycorrhizal symbiosis on plant viral diseases. The authors proposed the term “Mycorrhizal-Induced Susceptibility” (MIS) to describe the enhanced viral infection reported in many tripartite interactions (plant-mycorrhiza-virus), in opposition to the Mycorrhizal-Induced Resistance (MIR) that is often observed in response to infection by bacterial and fungal pathogens. Finally, Jones et al. summarized recent research that expands upon the role of keystone microbial species, phytohormones, and abiotic stress and how they relate to plant driven dynamic microbial structuring.

Plants are extremely plastic in their interaction with microorganisms, and the heterogeneous composition of the works published in this Research Topic well-represents the variety of responses, model and non-model organisms and experimental approaches used to investigate this subject. The knowledge of the determinants and the mechanisms that regulate plant-microbe interactions with different level of complexity can be instrumental for the development of new agro-biotechnological strategies of crop protection, with the aim to improve food security and environmental sustainability.

## Author Contributions

All authors listed have made a substantial, direct and intellectual contribution to the work, and approved it for publication.

### Conflict of Interest

The authors declare that the research was conducted in the absence of any commercial or financial relationships that could be construed as a potential conflict of interest.
